# Trend analysis in syphilis detection rates in pregnant women and in the incidence rate of congenital syphilis in the state of Ceará from 2015 to 2021

**DOI:** 10.1590/1980-549720230052.2

**Published:** 2023-12-01

**Authors:** Fabiola de Castro Rocha, Maria Alix Leite Araújo, Rosa Livia Freitas de Almeida, Ana Fatima Braga Rocha, Surama Valena Elarrat Canto, Ana Patrícia Alves da Silva

**Affiliations:** I Universidade de Fortaleza, Graduate Program in Collective Health – Fortaleza (CE), Brasil.

**Keywords:** Pregnancy, Congenital syphilis, COVID-19, Epidemiology, Gestação, Sífilis congênita, COVID-19, Epidemiologia

## Abstract

**Objective:**

To analyze the trend in the detection rate of Syphilis in Pregnant Women (SP) and in the incidence rate of Congenital Syphilis (CS) in the state of Ceará.

**Methods:**

This is an ecological study that used the technique of interrupted time series to analyze monthly data on cases of SP and CS obtained from the Department of Informatics of the Brazilian Unified Health System (DATASUS) from January 2015 to July 2021. The Kernel test and the Lincoln-Petersen estimate were used to analyze the statistical significance.

**Results:**

In 2015, a monthly detection rate of SP was estimated at 5.4 and a CS incidence rate of 8.2 per one thousand live births (LB). The implementation of the ordinance that changed the criteria for defining cases of SP and CS resulted in an increase of 4.9 (p<0.0001; 95%CI; 3.33; 6.61) in the detection rate of SP and a decrease of 0.1 on the incidence of CS (p<0.001; 95%CI -0.2; -0.1). The COVID-19 pandemic did not impact the monthly detection rate of SP (p=0.558; 95%CI 5.92; 3.22), nor its trend (p=0.7397; 95%CI 0.28; 0.3), but there was an increase of 0.19 in CS (p<0.001; 95%CI 0.1; -0.31).

**Conclusion:**

Between 2015 and June 2021, the trend in the detection rate of SP and in the incidence rate of CS was impacted by changes in the criteria for defining cases of these diseases proposed by the Brazilian Ministry of Health and the COVID-19 pandemic.

## RESUMO

**Objetivo:**

Analisar a tendência na taxa de detecção de sífilis em gestantes (SG) e na taxa de incidência de sífilis congênita (SC) no estado do Ceará.

**Métodos:**

Estudo ecológico que utilizou a técnica da série temporal interrompida para analisar os dados mensais de casos de SG e SC obtidos no Departamento de Informática do Sistema Único de Saúde (DATASUS) no período de janeiro de 2015 a julho de 2021. Utilizaram-se o teste de Kernel e a estimativa de Lincoln-Petersen para analisar a significância estatística.

**Resultados:**

Em 2015, estimou-se uma taxa de detecção mensal de SG de 5,4 e de incidência de SC de 8,2 por 1 mil nascidos vivos (NV). A implantação da portaria que alterou os critérios de definição de caso de SG e de SC implicou no aumento de 4,9 na taxa de detecção de SG (p<0,0001; IC95% 3,33; 6,61) e na queda de 0,1 na taxa de incidência de SC (p<0,001; IC95% -0,2; -0,1). A pandemia de COVID-19 não impactou na taxa de detecção mensal de SG (p=0,558; IC95% 5,92; 3,22), nem na sua tendência (p=0,7397; IC95% 0,28; 0,3), mas houve aumento de 0,19 na de SC (p<0,001; IC95% 0,1; -0,31).

**Conclusões:**

Entre os anos de 2015 e junho de 2021, a tendência na taxa de detecção de SG e na taxa de incidência da SC sofreu impacto das mudanças nos critérios de definição de caso desses agravos propostas pelo Ministério da Saúde e da pandemia de COVID-19.

## INTRODUCTION

The control of syphilis in pregnant women (SP) and congenital syphilis (CS) involves the quality of prenatal care, as well as the functioning and monitoring of health information systems, whose notification of cases and adequate completion of information enable the identification and planning of coping strategies. Since notification became mandatory for these diseases^1^, the Brazilian Ministry of Health (*Ministério da Saúde* – MS) has been adopting and modifying the definitions of cases in order to maintain the same standard of notifications throughout the national territory and align them with international criteria for comparison with other countries^2^.

In Brazil, as of 2017, the MS made important changes that may have had an impact on the detection rates of SP and incidence of CS. These changes occurred in relation to the notification and treatment of SP. Those who completed treatment up to 30 days before delivery were considered adequately treated; currently, the treatment is deemed adequate when starting up to 30 days prior to delivery. It was also suggested that SP should be reported during delivery or puerperium. Before, if the pregnant woman was not notified during prenatal care, she should be notified during delivery and puerperium as a case of acquired syphilis. Regarding the definition of CS case, the need to treat the sexual partner of the pregnant woman, who is infected with syphilis, ceased to be considered^3^. These changes have made case definitions less sensitive, and may decrease the number of diagnosed children and possible underreporting.

In 2020 and 2021, the new coronavirus (COVID-19) pandemic occurred, the largest in recent human history, an event that may have impacted the detection and incidence rates of SP and CS, respectively. COVID-19 has affected millions of people worldwide and killed 814,497 Brazilians in Brazil alone^4^. This pandemic has brought immeasurable consequences, including high costs for the health system with a drop in health care, especially in primary care. The state of Ceará ranked third in the highest number of reported cases in the country^5^.

The social isolation resulting from the COVID-19 pandemic required urgent measures to reorganize healthcare services, especially primary care, which practically suspended elective care and began to prioritize people with respiratory syndromes. Conversely, people have also stopped seeking these services for fear of exposure to the virus. These situations caused failures in diagnosis and treatment in primary care, with the interruption of the usual patterns of health care globally^6^.

Taking this into consideration, in this study we aimed to analyze the trend in the detection rate of SP and in the incidence rate of CS in the state of Ceará, Brazil, in the period from 2015 to 2021.

## METHODS

This is an ecological study that used the Interrupted Time Series (ITS) analysis technique to analyze the temporal trend in the detection rate of SP and in the incidence rate of CS in the state of Ceará in the period from 2015 to 2021. The state of Ceará has 184 municipalities and is geographically divided into five health macroregions, it is the fourth largest federative unit in the Northeast region, with a territorial area of 148,894.477 km² and an estimated population of 8,791,688 inhabitants, representing 4.33% of the population of Brazil in 2022^7,8^.

The detection rates of SP and incidence of CS were calculated from January 2015 to July 2021, whose data were obtained from the website of the Department of Informatics of the Brazilian Unified Health System (*Departamento de Informática do Sistema* Único *de Saúde* – DATASUS). These rates were calculated by the ratio between the number of cases reported each month for every one thousand live births (LB) of the same year and month for the study period.

ITS studies are used to evaluate population-based interventions and analyze the effects of a given intervention, considering a trend expected in its absence (counterfactual) and the trend found after the intervention (intervention). Comparing the expected trend without intervention and the trend after intervention enables to identify possible changes and thus assess their impact. This analysis strategy, considered as a quasi-experimental method, has strong internal validity, mainly because of its control over the effects of regression on the mean. The formula of Huitema and Mckean^9^ (Equation 1) is used for the regression model for a single group:


(1) $${\text{Υ}}_{\text{t}}{\text{= β}}_{0}{\text{+ β}}_{1}T_{\text{t}}{\text{+ β}}_{2}X_{\text{t}}{\text{+ β}}_{3}X_{\text{t}}T_{\text{t}}{\text{+ ε}}_{\text{t}}$$

Where:

Υ_t_ = outcome variable, aggregate measure at each equally spaced time point *t*;

*T*_t_ = time since the onset of the study;

*X*_t_ = dummy variable (indicator) representing the intervention (pre-intervention/event periods=0, otherwise=1);

*X*_t_ *T*_t_ = term of interaction between the time of the study and the intervention;

ε_t_: random error present in every statistical formula.

In the case of a single-group study, the following should be done:

β_0_ = represents the level of interception or onset of the outcome variable;

β_1_ = slope or trajectory of the outcome variable until the introduction of the intervention;

β_2_ = represents the change in the level of the outcome that occurs in the period immediately after the introduction of the intervention (compared to the counterfactual);

β_3_ = represents the difference between the pre-intervention and post-intervention slopes of the outcome.

Thus, the analysis focuses on significant p-values at β_2_, to indicate an effect of the immediate intervention, or at β_3_, to indicate an effect of the intervention over time^10^. In this study, the months of November 2017 and February 2018 were considered as time frames, to analyze, as interventions, the change in the case definition criteria of SP and CS, respectively, and the month of March 2020, to analyze the impact of COVID-19.

Initially, analyses were performed by simple comparison of the mean rates of the “before and after” periods using the Student’s t-test. Subsequently, the time series models for CS and SP were adjusted using the Stata/MP 16.1 software. Monthly rates were adjusted to account for differences in month length and were decomposed into trend components (the increasing or decreasing value) and seasonal components (the monthly seasonal pattern) using the LOESS (Locally Estimated Scatterplot Smoothing) methodology of seasonal trend decomposition.

When there was evidence of residual seasonality, adjustments were made to the seasonal window parameter until no residual pattern remained. The Newey-West procedure was used to calculate the standard error of the regression coefficients, which produces consistent estimates in the presence of autocorrelation, in addition to a possible heteroscedasticity^11^. Serial autocorrelation and heteroscedasticity were measured using the Cumby-Huizinga test^12^. The trend was calculated based on the Lincoln-Petersen estimate. A value of p<0.05 was considered statistically significant. The results were presented in tables and graphs with the values of coefficient, standard errors, trend, p-value, and confidence intervals (95%CI).

As these are secondary data in the public domain, in accordance with Decree No. 7,724/2012 and Resolution No. 510/2016, which regulate access to information and the rules applicable to research in public domain databases, respectively, the authorization of the research ethics committee was not required.

## RESULTS

**Figure 1.**
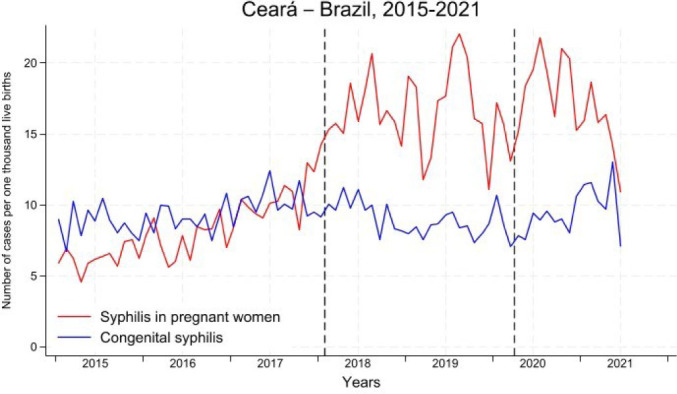
Syphilis detection rate in pregnant women and incidence of congenital syphilis. Ceará, January 2015 to July 2021.

Source: Department of Informatics of the Brazilian Unified Health System (DATASUS).

From January 2015 to July 2021, 10,562 cases of SP and 7,670 cases of CS were reported in the state of Ceará. In Figure 1 we show the detection rate of SP and incidence of CS. Between 2015 and 2017, we observed a higher incidence rate of CS in relation to the detection rate of SP. As of 2017, with the recommendation by the MS to notify SP at the time of delivery and puerperium, there was a considerable increase in the detection rate of SP. Regarding CS, notifications did not follow this increase, possibly due to the change in the CS case definition criterion, which withdrawn the need for treatment of the pregnant woman’s sexual partner.

The estimated monthly detection rate of SP at the beginning of the time series was 5.4 per one thousand LB (95%CI 4.5; 5.9), with a significant upward trend of 0.14 until October 2017 (p<0.0001; 95%CI 0.12; 0.20). The mean crude detection rate in the analyzed period was 7.9 per one thousand LB. In November 2017, the detection rate increased to 12.3 per one thousand LB, implying a significant increase of 4.9 (p<0.0001; 95%CI 3.33–6.61), tending to stabilize from that point on.

**Figure 2.**
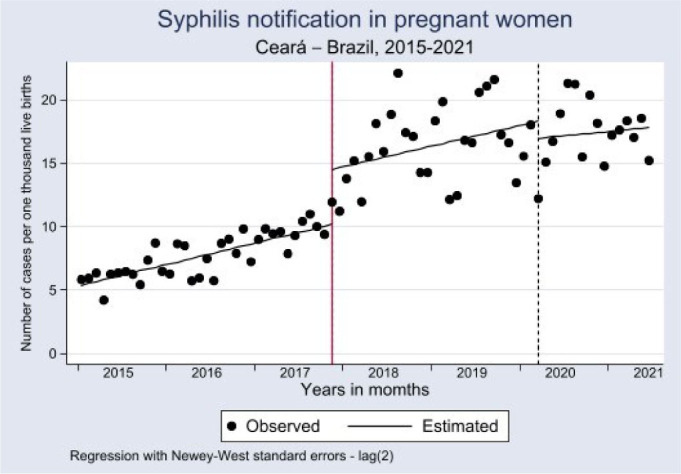
Interrupted time-series analysis of syphilis detection rates in pregnant women. Ceará, January 2015 to July 2021.

Source: Department of Informatics of the Brazilian Unified Health System (DATASUS).

The Lincoln-Petersen estimate produced by the post-trend specification showed a reduction of 0.01 from the previous trend, remaining stable, demonstrating that the ordinance increased the detection rate without, however, changing its trend. As for the COVID-19 pandemic, we observed no significant changes in the monthly detection rate (p=0.558; 95%CI 5.92; 3.22), nor in its trend in the evaluated period (p=0.7397; 95%CI 0.28; 0.3) (Table 1; Figure 2).

**Table 1:** Detection rate of syphilis in pregnant women. Ceará, January 2015 to July 2021.

SP Notification Estimates	Coef	Error*	t	p-value	95%CI
Time	0.14	0.01	11.49	<0.001	0.12; 0.17
Change in SP case criterion	4.35	1.29	3.37	0.001	1.78; 6.93
Interaction between change in SP case criterion and time	-0.01	0.09	-0.07	0.945	-0.18; 0.17
Pandemic	-1.35	2.29	-0.59	0.558	-5.92; 3.22
Interaction with the pandemic	-0.08	0.19	-0.41	0.686	-0.46; 0.31
Constant	5.39	0.24	22.37	0.000	4.91; 5.87
Trend after change in SP case criterion^†^	0.1349	0.0846	1.5947	0.1151	-0.03; 0.3
Post-pandemic trend^†^	0.0565	0.1694	0.3336	0.7397	-0.28; 0.39

Source: Department of Informatics of the Brazilian Unified Health System (DATASUS).

Coef: coefficient; 95%CI: 95% confidence interval; SP: syphilis in pregnant women; *Newey-West; ^†^Lincoln-Petersen estimate.

**Figure 3.**
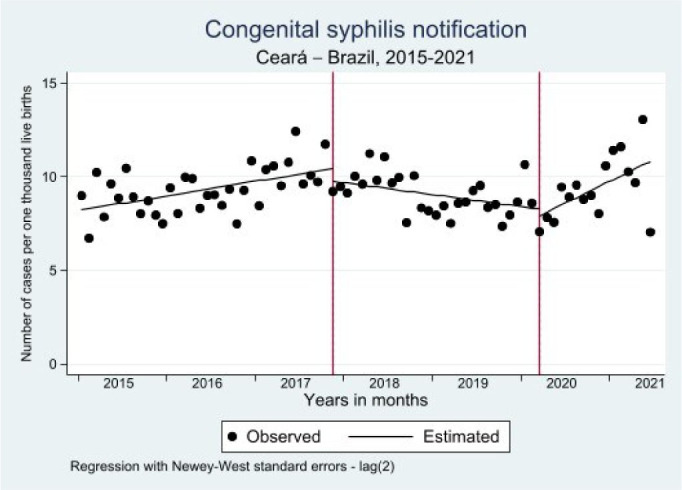
Interrupted time series analysis of congenital syphilis notifications. Ceará, January 2015 to July 2021.

Source: Department of Informatics of the Brazilian Unified Health System (DATASUS).

Regarding CS, an initial incidence rate of 8.2 per one thousand LB was estimated in 2015, indicating a significant increase of 0.07 in the monthly trend until 2021 (p<0.001; 95%CI 0.0; 0.1). In February 2018, after the publication of the information notice that changed the definition of the CS case, we identified no significant change in the monthly incidence rate. However, as of March 2017, there was a decrease of 0.1 notifications per one thousand LB in the monthly trend (p<0.001; 95%CI -0.2; -0.1) when compared to the period prior to the change in the case definition criterion. The Lincoln-Petersen estimate showed a decrease in the monthly trend of 0.06 in the incidence rate of CS per one thousand LB until April 2020 (p=0.0113; 95%CI -0.01; -0.02) (Table 2; Figure 3).

**Table 2.** Incidence rate of congenital syphilis. Ceará, January 2015 to July 2021.

Estimates	Coef	Error	t	p-value	95%CI
Time	0.07	0.02	3.74	<0.001	0; 0.11
Change in SC case criterion	-0.61	0.58	-1.05	0.297	-1.8; 0.55
Interaction between change in SC case criterion and time	-0.14	0.03	-4.34	<0.001	-0.2; -0.07
Pandemic	0.28	0.56	0.49	0.624	-0.8; 1.4
Interaction with the pandemic	0.26	0.06	4.09	<0.001	0.1; 0.39
Constant	-8.13	0.37	-22.24	<0.001	7.4; 8.86
Trend after change in SC case criterion*	-0.066	0.0254	-2.5986	0.0113	-0.1; -0.02
Post-pandemic trend*	0.1987	0.0556	3.5759	0.0006	0.1; 0.31

Source: Department of Informatics of the Brazilian Unified Health System (DATASUS).

Coef: coefficient; 95%CI: 95% confidence interval; CS: congenital syphilis; *Lincoln-Petersen estimate.

As of April 2020, the first month of the COVID-19 pandemic, we observed no significant change in the CS incidence in relation to the period after the change in case criteria. However, the Lincoln-Petersen estimate confirms a significant upward trend in these monthly rates of 0.19 (p<0.001; 95%CI 0.1; -0.31). The change in case criteria caused a reduction in the monthly incidence rates of CS of 0.06 per one thousand LB (p<0.011; 95%CI -0.01; -0.31), while the COVID-19 pandemic caused an increase of 0.19 per one thousand LB, with a significant difference of 0.26 (p<0.001; 95%CI 0.1; 0.39) monthly cases per one thousand LB (Table 2; Figure 3).

## DISCUSSION

In this study, we show that there was a significant increase in the trend in the detection rate of SP between January 2015 and July 2021 due to the change in the case definition criterion and reduction in the speed of the increase after the COVID-19 pandemic period. There was a decrease in the trend in the CS incidence rate after the change in the case definition and a worrisome subsequent increase. That is, even with a less sensitive case definition, there was an increase in the trend in the CS incidence rate during the pandemic period.

These findings corroborate data from the MS that show a constant increase in the detection rates of SP and incidence of CS, which ranged from 10.9 to 27.1 and from 6.6 to 9.9 per one thousand LB in the years 2015 to 2021, respectively^13^. Worldwide data that analyzed the incidence rate of CS between 2012 and 2016 showed a reduction in several regions of the world, except in the region of the Americas^14^. Brazil may be contributing to this high rate, considering that other factors may contribute to the increase in SP and CS such as the implementation of rapid testing in the prenatal routine and the growth of acquired syphilis in the population^13^.

In the search for studies in electronic databases on the topic of syphilis, there was a lack of information on the effects resulting from changes in the criteria for defining cases in notifications of SP and CS as well as the impact of the COVID-19 pandemic on these events. It should be noted that, because this is a very current topic and still ongoing, there are no robust studies that allow comparisons and counterpoints, making it difficult to compare this type of analysis.

The limitation of this study was that we analyzed secondary data, thus subjecting it to issues related to data incompleteness and underreporting. It is noteworthy that in order to minimize these effects and improve the quality of the data, linkage was performed between different databases. In addition, a trend analysis of the rates of SP and CS was not performed after the implementation of the rapid test for syphilis during pregnancy, considering the unavailability of records, as this indicator was not linked to the receipt of resources by the municipalities^15^.

We identified that in 2018 there was an increase in the detection rate of SP and a decrease in the incidence rate of CS, which may have occurred due to the change in the definition of CS case, which excluded the need for partner treatment as a notification criterion, and the recommendation not to notify the pregnant woman at the time of delivery as a case of acquired syphilis, but as SP, a situation that had been occurring until 2017^3^. National data show that between 2016 and 2017 there was a 15% increase in the incidence rate of CS (period before the change in the case definition), while in 2017 and 2018 (period after the change) this increase was only 6%^16^.

The recommendation to notify pregnant women with syphilis at the time of delivery/curettage or puerperium may have been the reason why there was no impact on the reduction in the incidence rate of CS, in addition to demonstrating loss of opportunity for treatment of pregnant women with syphilis and prevention of transmission of the infection to the baby. However, it was an important recommendation, considering that it gave greater visibility to the SP issue. The notification of the case only at the time of delivery or puerperium resulted in a lot of underreporting of cases. An increase in the detection rate of SP demonstrates the need to improve the quality of prenatal care and to adapt public policies to prevent and combat CS^17^.

When the notification of pregnant women was carried out only during prenatal care, in some Brazilian capitals, the detection rates of SP were lower than the incidence rates of CS. With the new recommendation, the detection rates of SP showed a significant increase in relation to the incidence of CS^13^, which was observed in this study. It is worth noting that implementing rapid testing for syphilis in the first prenatal visit may also have contributed to the increase in the detection rate of SP.

In 2018, the ratio between the national detection rate of SP and the incidence of CS was 2.4 pregnant women with syphilis for one child with CS. The Federal District and 14 other states — eight in the Northeast Region (including the state of Ceará), one in the South, two in the Southeast, one in the North, and two in the Midwest — had a ratio lower than the national level^16^. In the state of Ceará, while in 2016 and 2017 there was an increase in the CS incidence rate of 12%, a reduction from 10.2 to 9.6 per one thousand live birth was observed in the following biennium^16^. In addition, we observed that the incidence rate of CS is far from the target agreed by the World Health Organization (WHO) and the Pan American Health Organization (PAHO), which should be less than 0.5 cases per one thousand LB^18,19^.

It should be noted that according to the PAHO report, in 2017, of the 15 countries that submitted data indicating the elimination of CS, seven received an elimination certification by the WHO. Nonetheless, this agency highlights that CS cases were on the rise in 37 other countries, with 22% more cases this year compared to those recorded in 2016^18^. In Brazil, 43 cities were certified that they have reached the goal established by the WHO for the elimination of mother-to-child transmission of HIV and syphilis^20^.

With regard to the COVID-19 pandemic, there are indications that, worldwide, the combined effects of pressure on the health system and social lockdown, as a measure to reduce the transmission of the disease, are pointed out as a cause of global delay in diagnoses of sexually transmitted infections (STIs), including SP, due to the decrease in testing of the population, resulting from the interruption of the usual patterns of health care in primary care, which became overloaded with the care of patients with respiratory symptoms as of March 2020, as evidenced in research conducted in the United Kingdom, Spain, the United States, and Brazil^21-23^.

In a time-series analysis study of STI notifications between August 1st, 2017 and August 1st, 2020 in Spain, a reduction of almost 50% in notifications in that region was evidenced. Syphilis had 22% fewer notifications, followed by chlamydia, with a reduction of 28%^24^. A study whose authors analyzed the notification rates of SP in Brazil in the previous period and during the pandemic showed a 51.4% reduction in cases in the years 2020 and 2021, with important differences by federative units, with the North and Northeast regions showing the greatest reductions^25^.

This sharp decline observed in notifications during the COVID-19 pandemic could hardly be explained without considering the possibility of significant underreporting, the effective drop in the detection rate of acquired syphilis, possibly due to social isolation, the temporary barriers that hindered access to prenatal care, the reduction in routine consultations and exams, the prioritization of emergencies, and the exacerbation of social inequities during the pandemic^23^.

However, as demonstrated in this study, Ceará had an ascending detection rate of SP, signaling the commitment and care of professionals in reporting cases even in the face of adverse situations, such as work overload and consequent physical and emotional exhaustion, identified among health workers during the COVID-19 pandemic^22,26^. It is worth considering that since 2014 there has been an increase in the implementation of the rapid test for syphilis in the first prenatal visit, which may also have contributed to this rate.

The magnitude of the occurrence of vertical transmission of syphilis shows that this epidemic is far from being controlled, becoming more severe when it overlaps with another epidemic with a high morbidity and mortality rate, such as COVID-19, responsible for the recent collapse of the health system, a fact that imposes a great challenge for health managers and professionals regarding the effectiveness of actions to control and eliminate CS and its consequences.

All in all, we can conclude that between 2015 and June 2021, the trend in the detection rate of SP and in the incidence rate of CS was impacted by changes in the criteria for defining cases of these diseases, proposed by the MS, and the COVID-19 pandemic.
